# Quality and Nutritional/Textural Properties of Durum Wheat Pasta Enriched with Cricket Powder

**DOI:** 10.3390/foods9091298

**Published:** 2020-09-15

**Authors:** Marina Carcea

**Affiliations:** Research Centre for Food and Nutrition, Council for Agricultural Research and Economics (CREA), Via Ardeatina 546, 00178 Rome, Italy; marina.carcea@crea.gov.it; Tel.: +39-06-51494638

**Keywords:** edible insects, insect-based food, cricket powder, durum wheat semolina dry pasta, nutritional properties, technological properties

## Abstract

Edible insects have always been consumed by humans and nowadays they are looked at with interest by the research community as a means to produce food at low environmental cost for a growing and increasingly demanding population. A large number of different species are edible, and they can contribute fats, protein, fibre, vitamins, and minerals to the human diet. The absence of specific legislation on the use of insects as food, coupled with the general population’s disgust at the idea of eating insects, are among the limiting factors for the development of insect farming in developed countries. Several consumer studies have concluded that hiding insects in traditional foods can increase people’s willingness to eat insect-based foods. Cereal-based foods such as bread, bakery products, pasta, etc., being so popular worldwide and so widely accepted by the population, have been used by researchers as a carrier for the introduction of different percentages of insect flours to improve their nutritional qualities. The research by Duda et al. on “Quality and Nutritional/Textural Properties of Durum Wheat Pasta enriched with Cricket Powder” is the first recent scientific contribution to the understanding of the nutritional quality and technological effects of the introduction of insect flour in a popular food such as durum wheat pasta.

Edible insects have always been consumed by humans: nowadays people globally do not eat insects as a famine resource but out of choice because of their palatability and established place in local cultures. However, in most Western countries entomophagy is associated with primitive behavior.

In recent times insects have attracted researchers’ attention as a source of food for a growing and increasingly demanding population at low environmental cost. The wide use of insects as food involves the safe artificial rearing of insect species, the processing of insects into food, the labeling and marketing of insect-based foods, and consumers’ acceptance of this kind of food.

Considering worldwide consumption, the most commonly consumed species belong to the beetle group (Coleoptera) (31%), followed by caterpillars (Lepidoptera) (18%); bees, wasps and ants (Hymenoptera) (14%); grasshoppers, locusts and crickets (Orthoptera) (13%); cicadas, leafhoppers, plant hoppers, scale insects and true bugs (Hemiptera) (10%); termites (Isoptera) (3%); dragonflies (Odonata) (3%); flies (Diptera) (2%); and other orders (5%) [1]. Most edible insects are harvested in the wild and the concept of farming them for food is relatively new: cricket farming is a good example of this new enterprise.

Insects can be considered a nutritious and healthy food contributing beneficial fats, protein, fibre, vitamins and minerals, calcium, iron, and zinc in particular, to the human and animal diet. Nutritional value depends on the species, the metamorphic stage of the insect, the environment in which it grows, its diet and the way it is processed after harvesting. For example, Xiaming et al. [2] evaluated the protein content of 100 species from several insect orders and found that protein content ranged from 13%–77% of dry matter. Insects are often consumed whole, but they can be processed into flour, granular or paste forms and incorporated into other foods. Components such as chitin, proteins, fats, and oils can also be extracted from insects and used in foods [3,4]. Moreover, each insect has its own flavor which makes it suitable to be added to a specific food [5].

The absence of specific legislation on the use of insects as food, coupled with the general population’s disgust at the idea of eating insects, are among the limiting factors for the development of insect farming to supply the food industry in developed countries, even if the idea of novel foods can attract some consumers, especially the younger [6].

Some research groups have recently investigated the acceptability of foods containing insects in different countries: they concluded that providing information about the benefits of insect-eating, and using familiar foods, e.g., hiding insects in traditional foods, can increase willingness to eat this kind of food [7,8,9,10,11,12].

Cereal based foods such as bread, biscuits, bakery products, pasta, etc., being so popular worldwide and so widely accepted by the population, have been used by researchers as a carrier for the introduction of different percentages of insect flours and to study the products’ quality. Different insect species were introduced in the preparation of bread and bakery products, and preliminary results indicate that insect flours could open new alternatives for developing bakery products [13,14,15,16,17,18,19,20]. An interesting feature of insect powders in connection with bakery products is their possible use as a new protein source for gluten-free products where gluten proteins must be replaced by other sources [12,21,22]. 

Pasta is a convenient, nutritious food, easy to prepare and therefore widely consumed worldwide. Pasta has a long-standing research history of partial or total substitution of the raw material of choice, i.e., durum wheat semolina, with flours from other cereals or plants and with raw materials from animal sources. The reasons for these attempts include the need to improve the nutritional quality of wheat, in particular its protein quality, to use local raw materials, or to experiment with ancient or new cereal species such as triticale [23]. Insect flours, given their chemical composition, and in particular the aminoacidic composition of their proteins, could be good candidates to improve the nutritional quality of pasta. In fact, cereal proteins are generally low in lysine and in some cases lack the amino acids tryptophan (as in maize) and threonine. In some insect species, these amino acids are very well represented [1].

Studies on the fortification of pasta products with edible insects have appeared only recently in scientific literature: they are few [24,25,26] and none of them involved durum wheat semolina dry pasta. In these studies, millet flour blends, composite flours, and buckwheat flour were used to produce the pasta to be integrated with different insect powders.

The research study by Duda et al. on “Quality and Nutritional/Textural Properties of Durum Wheat Pasta enriched with Cricket Powder” [27] is a recent, interesting and high quality scientific contribution to the understanding of the nutritional qualities and technological effects of the introduction of insect flour in a popular product consumed worldwide, such as durum wheat pasta. To our knowledge, no other papers have been published so far on this specific topic so its publication is welcomed by the scientific community and by the food industry, which is keen to know more on the potential uses of insect flours in traditional foods. 

The authors used commercial cricket powder to enrich durum wheat semolina, which was then used to produce dry pasta. Three levels of semolina replacement were chosen: 5%, 10% and 15%. The products obtained were then analyzed for their nutritional composition, cooking, textural properties, color, and consumer acceptance. The results indicate that the addition of cricket powder influenced cooking weight and cooking loss (reducing losses and water absorption), as well as the color of pasta, reducing its lightness and shifting color balances to blue and red. The firmness of pasta was also influenced, the texture being strengthened by the addition of the cricket powder. Principal components analysis indicated that the flavor change had the most pronounced effect on consumer acceptance. Nevertheless, sensory evaluation proved that protein-enriched pasta produced with cricket powder gained a level of consumer acceptance comparable with that of conventional products. As expected, the addition of cricket powder produced a change in the pasta’s nutritional quality: protein content increased from approximately 10% to approximately 17% and fat and mineral content also increased significantly. 

In conclusion, we can say that more research is needed on the technological, nutritional and safety aspects of foods containing insects in order to promote the use of insects in foods and mainstream acceptance of such products.
